# Supplementary material for: Low-Density Lipoprotein Cholesterol and the Risk of Rheumatoid Arthritis: A Prospective Study in a Chinese Cohort
Source: Nutrients. 2022 Mar 15;14(6):1240. doi: 10.3390/nu14061240 (PMC8954206; doi:10.3390/nu14061240)
Supplement: Supplementary file 1 [file nutrients-14-01240-s001.zip › Supplementary Table S1.pdf]

Table S1a. Baseline characteristics in 2006 by high density lipoprotein cholesterol concentration, among 99,139 Kailuan participants without rheumatoid arthritis, adjusted for age and sex[a]

	High Density Lipoprotein Cholesterol			
	< 1.28 mmol/L	1.28-1.50 mmol/L	1.51-1.77 mmol/L	≥ 1.77 mmol/L
N	24319	25321	24739	24725
Women, %	16.3	18.8	21.8	23.5
Age, year	49.7±0.09	50.0±0.08	50.2±0.08	53.0±0.08
Alcohol intake, grams/day	3.69±0.16	4.85±0.16	5.35±0.16	7.52±0.16
Smoking status, %				
Never	55.5	57.0	62.6	63.5
Past	6.61	6.71	5.46	5.00
Current	37.9	36.3	32.0	31.5
CRP[b], mg/L	2.63±0.04	2.54±0.04	2.37±0.04	2.28±0.04
BMI[c], kg/m^2	25.4±0.02	25.2±0.02	24.8±0.02	24.4±0.02
Diabetes status, %				
Normoglycemic	69.0	68.5	71.8	71.5
Pre-diabetic	21.4	22.4	19.6	19.1
Diabetic	9.55	9.04	8.61	9.35
Hypertension status, %				

Normotensive	20.9	20.7	20.2	17.1
Pre-hypertensive	49.4	48.5	48.3	48.6
Hypertensive	29.7	30.7	31.5	34.3
LDL-C, mmol/L[c]	2.29±0.00	2.37±0.01	2.31±0.01	2.23±0.01
HDL-C, mmol/L[d]	-	-	-	-
Triglycerides, mmol/L	1.68±0.01	1.60±0.01	1.56±0.01	1.60±0.01

[a] Continuous variables are presented as the mean ± standard error, adjusted for sex and age. High density lipoprotein cholesterol concentrations are split into quartiles; 35 participants did not have data on HDL-c at baseline. Age is only adjusted for sex.

[b] C-reactive protein

[c] Body mass index

[d] Low density lipoprotein cholesterol (LDL-C)

[e] High density lipoprotein cholesterol (HDL-C)

Table S1b. Baseline characteristics in 2006 by triglyceride and total cholesterol concentrations, among 99,139 Kailuan participants without rheumatoid arthritis, adjusted for age and sex[a]

	Triglycerides			Total Cholesterol		
	< 1.02 mmol/L	1.02-1.65 mmol/L	≥ 1.65 mmol/L	< 4.51 mmol/L	4.51 – 5.34 mmol/L	≥ 5.34 mmol/L
N	32870	33409	32798	32942	33281	332899
Women, %	23.6	19.6	17.2	19.7	20.2	20.4
Age, year	50.2±0.07	51.3±0.07	50.9±0.08	49.4±0.07	50.7±0.07	52.1±0.07
Alcohol intake, grams/day	5.27±0.14	4.51±0.14	6.63±0.14	3.27±0.14	4.69±0.14	8.27±0.14
Smoking status, %						
Never	60.4	62.0	56.5	61.4	61.4	56.2
Past	5.66	5.85	6.33	6.06	5.44	6.34
Current	34.0	32.1	37.2	32.5	33.2	37.5
CRP[b], mg/L	2.44±0.04	2.37±0.04	2.54±0.04	2.54±0.04	2.42±0.04	2.39±0.04
BMI[c], kg/m^2	23.6±0.02	25.0±0.02	26.2±0.02	24.6±0.02	24.8±0.02	25.3±0.02
Diabetes status, %						
Normoglycemic	77.7	70.7	62.4	75.2	71.9	63.6
Pre-diabetic	17.2	21.0	23.7	17.6	20.2	24.2
Diabetic	5.08	8.35	13.9	7.25	8.00	12.2
Hypertension status, %						

Normotensive	28.8	17.9	12.6	23.8	19.6	15.9
Pre-hypertensive	48.9	49.4	47.8	48.6	49.7	47.7
Hypertensive	22.3	32.7	39.6	27.6	30.7	36.4
LDL-C, mmol/L[c]	2.21±0.01	2.38±0.01	2.31±0.01	1.94±0.01	2.25±0.01	2.72±0.01
HDL-C, mmol/L[d]	1.58±0.00	1.57±0.00	1.54±0.00	1.45±0.00	1.56±0.00	1.69±0.00
Triglycerides, mmol/L	-	-	-	1.44±0.01	1.46±0.01	1.91±0.01

[a] Continuous variables are presented as the mean ± standard error, adjusted for sex and age. Triglyceride and total cholesterol concentrations are split into tertiles; 62 participants did not have data on triglycerides, and 17 participant did not have data on total cholesterol, at baseline. Age is only adjusted for sex.

[b] C-reactive protein

[c] Body mass index

[d] Low density lipoprotein cholesterol (LDL-C)

[e] High density lipoprotein cholesterol (HDL-C)
