# Supplementary material for: Low-Density Lipoprotein Cholesterol and the Risk of Rheumatoid Arthritis: A Prospective Study in a Chinese Cohort
Source: Nutrients. 2022 Mar 15;14(6):1240. doi: 10.3390/nu14061240 (PMC8954206; doi:10.3390/nu14061240)
Supplement: Supplementary file 1 [file nutrients-14-01240-s001.zip › Supplementary Table S2.pdf]

Table S2. Adjusted hazard ratio and 95% confidence interval for risk of rheumatoid arthritis by blood lipid concentrations

Blood Lipid	# of case / population	Incidence rate (/10000 person-years)	Sex and age-adjusted hazard ratio	Multivariate-adjusted[a]	Multivariate-adjusted and hs-CRP adjusted[b]	Multivariate-adjusted last updated[c]	Multivariate-adjusted last updated and hs-CRP adjusted[d]
**High Density Lipoprotein Cholesterol**							
< 1.28 mmol/L	22/24660	1.12	1.00 (Ref.)	1.00 (Ref.)	1.00 (Ref.)	1.00 (Ref.)	1.00 (Ref.)
1.28-1.50 mmol/L	23/25581	1.11	0.96 (0.53, 1.72)	0.95 (0.53, 1.72)	0.98 (0.54, 1.78)	1.52 (0.84, 2.74)	1.53 (0.84, 2.78)
1.51-1.77 mmol/L	24/24959	1.19	0.98 (0.55, 1.75)	0.95 (0.53, 1.69)	0.95 (0.53, 1.73)	0.94 (0.49, 1.82)	0.98 (0.50, 1.90)
≥ 1.77 mmol/L	29/25006	1.45	1.00 (0.57, 1.77)	0.96 (0.50, 1.69)	0.91 (0.50, 1.67)	1.05 (0.56, 1.95)	1.12 (0.60, 2.10)
P_{trend}			0.95	0.98	0.98	0.61	0.82
**Triglycerides**							
< 1.02 mmol/L	32/33052	1.20	1.00 (Ref.)	1.00 (Ref.)	1.00 (Ref.)	1.00 (Ref.)	1.00 (Ref.)
1.02-1.65 mmol/L	38/33702	1.41	1.16 (0.72, 1.86)	1.23 (0.76, 1.99)	1.20 (0.74, 1.95)	0.80 (0.49, 1.29)	0.84 (0.52, 1.36)
≥ 1.65 mmol/L	29/33430	1.08	0.86 (0.52, 1.43)	0.93 (0.54, 1.58)	0.83 (0.48, 1.45)	0.64 (0.36, 1.13)	0.68 (0.38, 1.21)
P_{trend}			0.44	0.58	0.50	0.21	0.20
**Total Cholesterol**							
< 4.51 mmol/L	27/28598	1.19	1.00 (Ref.)	1.00 (Ref.)	1.00 (Ref.)	1.00 (Ref.)	1.00 (Ref.)
4.51 – 5.33 mmol/L	51/48840	1.29	0.94 (0.57, 1.55)	0.95 (0.57, 1.56)	0.98 (0.59, 1.63)	0.90 (0.54, 1.52)	0.92 (0.54, 1.54)
> 5.33 mmol/L	21/22517	1.16	1.07 (0.66, 1.73)	1.10 (0.67, 1.79)	1.12 (0.68, 1.84)	0.95 (0.57, 1.59)	1.03 (0.61, 1.72)
P_{trend}			0.77	0.74	0.63	0.86	0.89

[a] Adjusted for sex, age, body mass index (< 18.5 kg/m^2, 18.5- 23 kg/m^2, 23-27.5 kg/m^2, > 27.5 kg/m^2), anti-hypercholesterolemia drug use (yes, no), high density lipoprotein cholesterol (HDL-C) (quartiles), low density lipoprotein cholesterol (LDL-C) (tertiles), triglycerides (tertiles), alcohol consumption: never or past, light to moderate (women: 0–1.0 servings/d; men: 0–2.0 servings/d), and heavy (women: >1.0 serving/d; men: >2 servings/d), smoking (never, past, current), diabetes (non-diabetic, pre-diabetic, diabetic) and hypertension (no hypertension, pre-hypertension, hypertension)

[b] Adjusted for above covariates and further adjusting for high sensitivity c-reactive protein (hs-CRP) (< 1 mg/L, 1-3 mg/L, >3 mg/L)

[c] Adjusted for above covariates, using the most recent measurements before end of follow up, death, or rheumatoid arthritis diagnosis

[d] Adjusted for above covariates, using the most recent measurements before end of follow up, death, or rheumatoid arthritis diagnosis and further adjusting for high sensitivity c-reactive protein (hs-CRP) (< 1 mg/L, 1-3 mg/L, >3 mg/L)
